# Book Notices

**Published:** 1866-09

**Authors:** 


### EDITORIAL.

### BOOK NOTICES.

*Clinical Lectures by Prof. A. von Graefe, on Amblyopia and Amaurosis, and the Extraction of Cataract.* Translated by H. DERBY, M. D., Surgeon to Massachusetts Charitable Eye and Ear Infirmary, etc., etc.

Modern science has devised such improved means of investigating diseases of the eye, that our profession is now able to determine with great accuracy a diagnosis, where formerly all was doubtful.

The terms amblyopia and amaurosis, for instance, but a few years since, embraced several diseases, which can now be easily recognized as entirely distinct affections.

These advances in accuracy of diagnosis are not only of great interest to the specialist as a matter of pure science, but often of great practical advantage to the patient. We are able to give a favorable prognosis and to benefit our patients in cases which were considered as hopeless by the older oculists.

On the other hand, we are able with more confidence than ever to declare certain cases as positively incurable, thus removing painful suspense, and saving the patient the perplexity of seeking relief in vain.

The ophthalmoscope discloses with absolute certainty many abnormal conditions of the vitreous humor, of the choroid, of the retina and optic nerve, which were all formerly described more or less loosely as amaurosis.

The labors of Donders in resuming and extending the investigations of Airy and Young, have also eliminated from the so-called amaurotic diseases, another abnormal condition—astigmatism, or certain irregularities of curvature in the surface of the cornea or lens, by which rays of light are so diffracted, that the images on the retina are always more or less indistinct. Such eyes were regarded as amblyopic, and proved incurable by any means known a few years since. Now by the simple use of cylindrical glasses, patients with this defect, are enabled to see distinctly.

The intimate connection of the nerves of vision with the brain, has interested those who are devoting their labors to general nervous affections, in investigating with the ophthalmoscope the abnormal conditions of the retina and optic nerve.

Although something has been accomplished in this direction, there are many isolated, unexplained facts as regards the relation of certain symptoms in affections of the optic nerve with the brain and spinal cord.

We may expect that future investigations will solve many of these mysteries.

Dr. Derby has performed a valuable service for the profession in thus presenting to the American reader a good translation of these most admirable clinical lectures of von Graefe.

Our readers generally, who have not been particularly interested in ophthalmology, have but a faint idea, we believe, of the genius of von Graefe, of the extent of his labors, and the facilities he possesses for observation.

The number of the important operations of every kind on the eye which he has performed, might seem almost incredible to men in general practice.

From his enormous practice, both private and in hospital, he has a vast amount of material which he appropriates for the benefit of science, constantly employing as his assistants in his investigations men thoroughly educated, each in his own department of pathology, ophthalmoscopy and microscopy.

His extraordinary talents as a student of science, and as an observer of the phenomena of disease in an almost innumerable aggregate of cases, has made him one of the most remarkable men in the medical profession.

It is most worthy of notice, that while von Graefe has for several years devoted his talents to ophthalmology, he so devoted himself in his early career to the whole range of medical science, that he was capable, as has been stated, of acting as a proficient specialist in any department of medicine and surgery.

The first twenty-four pages of this little work contain a discussion of the general subject of amaurosis. These are followed by a most critical analysis of eight interesting cases.

We would especially commend to our readers, in any way interested in the subject of cataract, the series of clinical remarks on the extraction of cataract which form the conclusion of this pamphlet, and which, "as a specimen of clear, precise and comprehensive clinical instruction, the translator believes to be without a parallel in ophthalmic literature."

We can extract but a few passages on amaurosis, giving in full the report of the first case.

Experience has abundantly shown these forms which tend to progressive blindness to be characterized by an early narrowing of the field of vision, a preponderating loss of sensibility of the peripheric portions of the retina. It is, *a priori*, readily imaginable that, in a progressing atrophy of the nervous elements, those regions should first suffer which are most remote from the nutritive and functional centre, and that thus the extinction of power should be successive and, to a certain extent, centripetal. Inasmuch, therefore, as extreme significance is to be attached to a defective or diminished peripheric vision, our methods of diagnosticating the same must be as perfect as possible. Ordinary daylight is insufficient to detect slight defects in making a general examination of the periphery of the field of vision. This must rather be conducted in a darkened room where light proceeds from but a single source. Where absolute accuracy is desirable, the "graduated lamp" may be used; the diaphragm being set at 100, and a black paper without gloss being held before the patient (of course at a fixed distance.) The limits of the field of vision are ascertained by means of white balls, set on a black rod and gradually removed from the point of fixation. To ascertain the angle of distinction in eccentric vision, the balls may be placed on the two extremities of a blackened pair of compasses.

Especially interesting is the connection of progressive amaurosis with paralysis and mental alienation, and thus with gray regeneration of the spinal cord. It is well known that amaurotic affections not infrequently supervene on disordered states of the intellect; but sufficient stress has not been laid upon the fact that a large number of those attacked with amaurosis, who were in perfect possession of their faculties when first affected, are subsequently the victims of dementia. While, therefore, amaurosis may not infrequently be looked upon as a premonitory symptom of mental derangement, the reverse is almost without exception the case in gray degeneration of the spinal cord. Characteristic symptoms of the spinal affection (impairment of sensibility) have become settled before the advent of the amaurotic affection. This may be explained by the anatomical fact that the course of the degeneration is from the vertebral column towards the interior of the skull. Of all the many cases of spinal amaurosis (forming, as they do, some thirty per cent. of the graver forms of progressive amaurosis) which came under my observation, I can recollect but two instances where the disease had progressed in an opposite direction. In the one the amaurotic long preceded the spinal affection, the patient having been entirely blind five years before the first eccentric pains, followed by the usual signs of tabes, made their appearance. In the other, the amaurotic affection had also lasted several years, but at the time of occurrence of the spinal disease there remained some perception of light in one eye.

Graefe's opinion regarding the use of tobacco as a cause of amaurosis is worthy of consideration.

The fact which cannot be denied, that amblyopia affects men more frequently than women, the proportion with us being about 4 to 1, has seemed to justify the conclusion that smoking is one of the chief causes; many of the other conditions, however, are especially applicable to men, and, in my opinion, excessive smoking must be regarded, in the majority of cases, as simply among the active causes.

CASE I. *Curative form of Congestive Amblyopia, with Normal Field of Vision.*

Florian M., railway employee, æt. 49, of healthy appearance, cheeks and extremity of nose of rather a venous redness, comes on account of impaired vision of both eyes. This has been creeping on for the last twenty months; at first very gradually, during the last four months, however, has perceptibly increased. The functional examination shows that the acuteness of vision (tried by average daylight) has fallen in the right eye to $\frac{1}{6}$, in the left to $\frac{1}{7}$. The periphery of the field of vision (examined by softened lamplight) proves to be *absolutely normal*, and in accordance with this there can be found neither interruptions, misty spots, nor breaks in the acuteness of eccentric vision. Inspection discovers nothing abnormal about the exterior or interior of the eye; indeed, in spite of the already considerable duration of the disease, the papilla retains its delicate red, semi-transparent color.

Its nasal segment is appreciably more reddened than the temporal, owing to the greater number of small vessels and the thicker layer of nerve-fibre in the first direction. To this may be added that the inner is not so sharply defined as the outer edge of the choroid, still a careful inspection reveals the whole contour. Such a condition of the papilla may be regarded as entirely physiological, provided nothing abnormal be found in the vessels and the tissue. It is more or less marked, according to natural varieties in the optic nerve, and shows best generally in a case of physiological excavation. The larger veins are well filled, but are not abnormally tortuous, either as regards their own axis or the plane of the retina. We cannot, then, consider this as a symptom of disease; all the less, in fact, because the patient's complexion bears evidence of habitual venous engorgement.

An investigation of the case shows that the patient has for a long time indulged moderately in brandy, drank large quantities of beer, smoked considerably, and had his rest broken by the duties of his calling. He has suffered from neither digestive nor cerebral disturbance, and enjoyed good health. The result of the examination of the organs of respiration and circulation, the pelvic viscera, the skin and the urine is negative.

As regards the *prognosis* of this case, we may venture on an opinion in every respect favorable. In the first place, a progressive blindness is not to be feared in the least; for in spite of the long continuance of the disease, the boundaries of the field of vision prove to be entirely normal. But we may even speak confidently of improvement and restoration of vision, for (1) the continuity of the field of vision is entirely uninterrupted; it is only an affair of general diminution of sensitiveness, no indication of a central scotoma being present; (2) the appearance of the optic papilla is unchanged; notwithstanding the difficulty has lasted two years, there is no trace of atrophic degeneration; (3) in the habits of life of the patient, we find palpable causes, amenable to therapeutic influences, and of a kind that experience teaches us may produce derangements capable, to a certain extent, of removal.

It would be a difficult task to accurately explain the *nature* of this affection. No signs of active cerebral congestion exist. The venous redness of the face and the predisposing causes render probable a so-called passive congestion; this expression, however, conveys but a limited amount of information. Suppose the case to be one of an inundation of the central nervous structures with venous blood, or of a want of rapidity in the movement and change of the blood itself, or let there be a diminished activity of function on account of the blood being overloaded with alcoholic and narcotic substances—every one of these suppositions would be explained on the ground of "passive congestion of the brain." The only general and satisfactory use of this term is, therefore, in cases where, there being no signs of active congestion, the functional as well as the nutritive activity of the cerebral centre of the optic nerve has been reduced by influences springing from the source alluded to and acting through the circulation.

How is the *cure* of this case to be effected? First, of course, by paying a proper regard to its cause. The use of alcoholic beverages must be given up, that of tobacco reduced to a minimum, regularity in diet and sleep insisted on. Not infrequently is this course alone sufficient to cause a gradual retrogression of the symptoms in cases of this form of amblyopia. Experience teaches us, however, that there are more efficient means of securing and hastening a favorable result. As regards local depletion, a rapid evacuation of the blood is here of special importance. I was some time since led by this to appreciate the application of the leech of Heurteloup in the treatment of congestive amblyopia, and the method based on this has found much favor with the profession.

A good deal depends here on the manner of application. If the operator to whom we commit the task is not competent to fill a cylinder in a few minutes, the particular advantage of this method is lost. The application must be made in the evening, so as to give the full benefit of the night's rest. It is, moreover, desirable, in many cases that the patient should preserve perfect quiet the next day and remain in a dark room. As the importance of this precaution in such case depends on a careful consideration of the individual circumstances, it is best to regard it as a general rule. It is of consequence on account of the excitability of the cerebral circulation, or—if preferred—of the vaso-motory nerves. In cases where this is considerable, each application is followed by a period of excitement, characterized by derangements of sensibility of every kind, sometimes by subjective appearances of light, and even by some diminution of acuteness of vision. This period of "reaction," lasting only in exceptional cases more than a day, should be prepared for by entire bodily rest and a strict seclusion from light. This state, moreover, is least marked in cases of amblyopia arising from passive cerebral congestion, and most so in certain affections of the choroid; the period of rest, therefore, is less indispensable in the former case than in the latter. From two to four ounces of blood should be taken at each application. And this should be repeated at intervals of four, six or eight days, according to the constitution of the patient and the duration of the period of reaction. A careful examination of the acuteness of vision instituted directly prior to and two days after the application (the period of reaction being thus passed) will decide the propriety of repetition. Where the acuteness of vision has not been affected by two, at the most by three applications, they should be omitted. But even where a perceptible effect is noticed, I do not advise a too frequent repetition in cases of passive cerebral congestion, although of course the state of the constitution must influence the decision in individual cases. For it has been ascertained that those cases which evince improvement after the first three or four bloodlettings, do better under diaphoretics than by a continuance of the abstraction of blood, and it is our duty—other things being equal—to give the first named the preference as a less severe course.

Be it said, in this connection, that the Heurteloup leech, rendering, as it does, most valuable service in congestive amblyopia and chronic affections of the choroid, is by no means as good as the natural one in the treatment of the different. forms of ophthalmia. It is here that we derive more advantage from a prolonged suction and a continuous flow of blood than from the rapidity with which it is lost. Although warmly recommending the Heurteloup leech in cases of amblyopia with passive cerebral congestion, I am willing to admit that some cases derive more benefit from other measures. This is especially the fact when the excitability of the circulation is extremely pronounced. Here the period of reaction is seen to be abnormally lengthened and indisposed to yield to the desired remission. In such cases a decidedly better result is often obtained from cupping in the neck, in hæmorrhoidal affections from leeches to the anus, and in disorders of menstruation from cupping on the inside of the thighs; indeed, under these circumstances the Heurteloup leech may even increase the disturbance and harm the case. The question so often raised in practice as to whether the removal of blood shall take place from the immediate vicinity or at some distance from the affected part, depends in a measure on the method employed, and especially on the degree of excitability, and considerable caution must be used in answering the question in the first sense. A common mistake in cases of ophthalmia is to place the leeches nearer the eye than consorts with the sensitiveness of this organ and the surrounding structures. Further individual circumstances may sometimes induce us to employ other applications in preference to the Heurteloup leech on the temple, as when the disease seems, for example, to be connected with the cessation of habitual epistaxis, or of a hæmorrhoidal flow, etc.

Our diaphoretic treatment was principally (in imitation of many older practitioners) carried out by means of the decoction of Zittmann, not because any specific effect is to be attributed to separate ingredients of this complex draught, but because experience has shown it to be an excellent diaphoretic of proved worth. The strong decoction, warmed, was therefore given at an early hour to the patient in bed, and its action on the skin assisted by means of woolen coverings and afterwards by elder-tea, while on the other hand no special restrictions with regard to diet, as is generally the custom when this preparation is used, were imposed on the patient; a walk, too, in the afternoon was allowed when the weather was favorable. Of late we have been more sparing in the employment of the decoction of Zittmann, having learned to recognize a thoroughly invaluable therapeutic agent in well-appointed Roman baths.

My first inducement to employ Roman baths in cases of amblyopia with passive cerebral congestion was furnished me by a patient who had been but moderately benefited by a course of decoction of Zittmann, and entirely cured himself of his amblyopia by remaining in the warm boiling-room of a sugar-factory the heat being nearly 40°, (122° Fahrenheit.) Great advantages as the Roman baths possess in most of these cases over the Russian baths, they have, of course, their contra-indications, among them the more active states of congestion, and especially cardiac and renal affections, an apoplectic tendency and undue excitability of the circulation.

[These "Roman baths" are known in this country as Turkish baths, and differ from the Russian in the employment of dry heat instead of vapor.—TRANSLATOR.]*

The patient was again presented at the clinique four weeks after the commencement of his treatment. His course of life had been regulated and the Heurteloup leech applied three times, in consequence of which his acuteness of vision had successively increased from $\frac{1}{6}$ on one side and $\frac{1}{7}$ on the other to $\frac{1}{4}$ on each. Following this, a Roman bath had been taken every three days. No result followed the first bath, except a headache which lasted twelve hours, owing to the fact that he had not remained long enough in the sweating room and had been disturbed by the application of a cold douche at the wrong time. The next baths, in the administration of which these points were attended to, produced an excellent result, the acuteness of vision having risen, after the filth had been taken, on the right to $\frac{2}{3}$, on the left to more than $\frac{1}{2}$. A speedy and entire recovery is therefore no longer doubtful.

Von Graefe remarks, in conclusion, as follows. Recovery does not follow as rapidly in all these cases of amblyopia as in the present. If, however, appropriate treatment succeeds in once causing the affection to take such a turn that the acute-

* During a recent visit at the East, we found medical men employing these baths with much advantage, where ordinary hot baths were usually recommended.—ED.

ness of vision begins to increase, we may reckon with nearly entire confidence on a gradual and almost spontaneous improvement, provided the cause of the difficulty continues to be avoided. The attempt must not, therefore, be made to perfect the result by continuing the employment of vigorous measures during a prescribed time, but rather, after the first blow has been administered to the disease, it should be left to itself awhile, after which the former treatment may be resumed. Thus the entire amount of previous vision may often be seen to return in the space of several months or more. On the other hand, a strong tendency to relapse may be manifesed, when owing to a faltering resolution or the pressure of circumstances the patients again come within the influence of the previous exciting cause of the disease; in fact, such relapses may exhibit a paralytic tendency, differing from the previously mild form, (as in the case of amblyopia potatorum.) This teaches us how necessary it is to lay particular stress on the manner of life of such patients and regulate their labor. Finally, be it remarked that while we very frequently employ the course that has here proved itself efficacious, it should by no means be regarded as the exclusive method of treatment in such forms of the affection. No cases need more individual study than those of amblyopia, and the physician who after a single and hasty survey undertakes to give such patients advice available for some time to come, commits a serious fault. The derangements in the circulation which are here concerned may spring from the most varied sources, and it is both perilous and narrow-minded to deduce them by preference from some particular organic affection, from the liver, for example, or the alimentary canal, or some irregular course of life. Abdominal disorders, it is true, often play a principal part among the causes, and in accordance with this view, we often see good effects resulting from the use of mineral springs, such as Marienbad, Kissingen, Homburg, and in suitable cases particularly Carlsbad; as a general thing, however—thanks to the prevailing tendency—the functions of the abdominal viscera occupy too exclusively the attention of the physician. The weighty functions of the skin and kidneys, so important in their effect on amblyopic affections, are among those seriously neglected through this preference. While the regularity and amount of the fæcal evacuations are anxiously dwelt upon, these principal regulators of the circulation are hardly regarded, as being of minor importance. Even the ways of life, from which but too often proceed the accumulated causes of derangement of the circulation, are often but carelessly scanned by the physician, and as a matter of course made light of by the patient. If the sleep be not particularly disturbed, little is said about it, and yet good and regular sleep affords the most grateful refreshment to the unceasingly active nerve of vision. To be here a successful physician, one must examine with extreme care and weigh the result with foresight and impartiality.

---

*Cholera: Facts and Conclusions as to its Nature, Prevention and Treatment.* By HENRY HARTSHORNE, A. M., M. D., etc., etc. Philadelphia: J. B. Lippincott & Co. 1866.

This is a pamphlet written by a man of positive convictions. We like to read the works of such men, even if compelled to dissent from their views. But in the present case there is much to commend. The author is a decided anti-contagionist, and is skeptical with reference to the portability of the cause of cholera. He does not believe that the disease is "a disorder simply of the stomach and bowels;" he sums up his whole theory of the nature of the disease in one brief aphorism—"Cholera is, then, I say, a poison-spasm; a ganglionic tetanus."

The causation of the disease is argued with much ingenuity. The conclusion finally reached is "that the cause of cholera is a (yet undiscovered) protozoon, or primal organism, of extreme individual minuteness; which, on entering the human body, affects it as an organic poison." Whatever name may be attached to this "primal organism," the fact of an entity which produces the disease can hardly be questioned. We cannot believe in what are called purely *dynamic causes* for such a train of symptoms as are presented by the cholera patient.

Our author disbelieves in the efficacy of quarantine, and insists "that Sanitary Police includes the sum total of available measures for the prevention of cholera in any place."

Respecting the great question of treatment, his remarks are generally consistent with his theory of the nature and the cause of the disease. To bleed he fears, and from calomel he would wholly abstain. The eliminative treatment, which has been advocated by Dr. Johnson, of London, finds no favor. But in his remarks upon the eliminative *theory*, he is neither fair nor consistent. Because vomiting and purging are eliminative methods, it does not follow that elimination is dangerous or undesirable. Nor is it, on the other hand, true that the advocates of an eliminative treatment are advocates of emetics and cathartics in cholera. While the other natural channels of elimination are obstructed, to neglect them for the administration of castor oil and mustard, is to do what was called by Prof. Gilman, "looking at the patient through a pin-hole." No method of treatment which ignores the necessity of restoring healthy *excretion* as well as secretion, can ever attain the highest success in the treatment of cholera.

The use of ice along the spine is by our author still regarded as merely experimental. Sulphuric acid receives a *quasi* recommendation, "being so potent a destroyer of everything organic." Opium in large doses is rejected, as it should be, and the doctor proclaims his adhesion to the method of "treatment by anti-spasmodics and mild stimulants, in small doses at short intervals; with ice, and external frictions." During the *rice-water* stage, he uses the following recipe:

℞ Chloroform, tr. opii, spts. camphor, spts. ammon. aromat., aa. f ʒj ss; creosot., gtt. iij; ol. cinnam., gtt. viij; spts. vin. gall. f ʒij—M. Dissolve a teaspoonful of this in a wine glassful of ice-water, and give of that two teaspoonfuls every five minutes; followed each time by a lump of ice. Better even than this we like the recipe of Dr. Aitken: ℞ Ol. anisi, ol. cajeput., ol. juniperi, acid. sulph. aromat., aa. f ʒ ss.; ether sulph., f ℥ ss; tr. cinnam., f ℥ ij.—M. Dose, ten drops in a tablespoonful of ice-water every fifteen minutes. With the first two doses, give some preparation of opium and camphor. During the stage of collapse, nothing is definitely recommended by Dr. H., unless it be the use of belladonna or atropine, "as an antagonist of vascular spasm."

Such, then, is a brief outline of this timely little pamphlet. Its perusal will injure no one, and if the consideration of its arguments can prevent that wholesale use of opiates and astringents which is still so popular in certain quarters, it will not have failed of its purpose.

---

**We have received a very complete Illustrated Catalogue of Surgical Instruments of Geo. Tiemann & Co.'s manufacture, issued by their agents, Messrs. Bliss & Sharp, 144 Lake street, who will furnish them to the profession on application.**

*Shakspeare's Delineations of Insanity, Imbecility and Suicide.* By A. O. KELLOGG, M. D., Assistant Physician, State Lunatic Asylum, Utica, N. Y. Hurd & Houghton, N. Y. 1866.

In this little volume are collected a number of essays which were published by their author in the *American Journal of Insanity*, at various intervals between 1859 and 1864. As indicated by the title-page, the morbid characters of Shakspeare are classified as insane, imbecile and suicidal. The victims of insanity are Lear, Macbeth, Lady Macbeth, Hamlet, Ophelia, Jaques and Cordelia. The imbeciles are Bottom, Dogberry, Elbow, Shallow, Malvolio, Launce, Caliban, good Bardolph and his two friends, Nym and Pistol. The suicide stands alone—Othello.

Dr. Kellogg has produced a very readable book. His familiarity with the varied phases of mental derangement enables him to speak with authority in criticism of character. One cannot fail to admire the wonderful genius of the great dramatist, as it is made to appear with additional brightness the more closely it is examined. Shakspeare probably thought not whether his heroes and princes were to be counted, labelled and ranked with the sane or the insane. Simple truth was all for which he cared. He put on paper what he saw; and the difference between other singers and their king consisted only in the clearness of his sight. Shakspeare was the seer from whom nothing was hid. He saw, and described as he saw, every varying shade of character; so that the beholder of his work is impressed only with its ease, its truth and its universality. Other men see dimly or superficially; and their pictures please by halves. "Yes—that is very good; people sometimes act as the author describes; so they appeared to his mind's eye; that passage is very natural," etc., etc., is the best praise that can be awarded to the majority of authors. It is only when we sit down to the works of the master that the scales drop from our eyes, and all the treasures of heaven and earth are opened to our gaze, and we think no more of the methods by which such glory becomes visible. Other writers give us images and sketches of life—Shakspeare gives us Life itself.

For sale by S. C. Griggs & Co., 41 Lake st., Chicago.

*Medical Diagnosis.* By J. M. DaCosta, M. D. Philadelphia: J. B. Lippincott & Co. 1866.

We are glad to see this new edition of an invaluable work. The student who wishes to learn the art of diagnosis, without which the science of therapeutics is almost useless, will here find a sure guide. The work has been thoroughly revised, and improved to correspond with the latest advances in the methods of detecting disease. The uses of the sphygmograph, the thermometer and the laryngoscope are fully detailed, while the latest discoveries of the neuropathists and the helminthologists are properly accredited. A more instructive volume cannot be put into the hands of the medical student. Its typographical execution is all that could be desired.

For sale by S. C. Griggs & Co., 41 Lake street, Chicago.

